# Differential adenine methylation analysis reveals increased variability in 6mA in the absence of methyl-directed mismatch repair

**DOI:** 10.1128/mbio.01289-23

**Published:** 2023-10-05

**Authors:** Carl J. Stone, Gwyneth F. Boyer, Megan G. Behringer

**Affiliations:** 1 Department of Biological Sciences, Vanderbilt University, Nashville, Tennessee, USA; 2 Biodesign Center for Mechanisms of Evolution, Arizona State University, Tempe, Arizona, USA; 3 Department of Pathology, Microbiology, and Immunology, Vanderbilt University Medical Center, Nashville, Tennessee, USA; University of Pittsburgh School of Medicine, Pittsburgh, Pennsylvania, USA

**Keywords:** experimental evolution, mutation, epigenetics, *Escherichia coli*, N^6^-methyladenine, epimutation

## Abstract

Methylated DNA adenines (6mA) are a critical epigenetic modification in bacteria that affect cell processes like replication, stress response, and pathogenesis. While much work has been done characterizing the influence of 6mA on specific loci, very few studies have examined the evolutionary dynamics of 6mA over long time scales. We used third-generation sequencing technology to analyze 6mA methylation across the *Escherichia coli* K-12 substr. MG1655 genome. 6mA levels were consistently high across GATC sites; however, we identified regions where 6mA is decreased, particularly in intergenic regions, especially around the −35 promoter element, and within cryptic prophages and IS elements. We further examined 6mA in WT and methyl-directed mismatch repair-deficient (MMR-) populations after 2,400 generations of experimental evolution. We find that, after evolution, MMR- populations acquire significantly more epimutations resulting in a genome-wide decrease in 6mA methylation. Here, clones from evolved MMR- populations display non-deterministic sets of epimutations, consistent with reduced selection on these modifications. Thus, we show that characterization of 6mA in bacterial populations is complementary to genetic sequencing and informative for molecular evolution.

**IMPORTANCE:**

Methylation greatly influences the bacterial genome by guiding DNA repair and regulating pathogenic and stress-response phenotypes. But, the rate of epigenetic changes and their consequences on molecular phenotypes are underexplored. Through a detailed characterization of genome-wide adenine methylation in a commonly used laboratory strain of *Escherichia coli*, we reveal that mismatch repair deficient populations experience an increase in epimutations resulting in a genome-wide reduction of 6mA methylation in a manner consistent with genetic drift. Our findings highlight how methylation patterns evolve and the constraints on epigenetic evolution due to post-replicative DNA repair, contributing to a deeper understanding of bacterial genome evolution and how epimutations may introduce semi-permanent variation that can influence adaptation.

## INTRODUCTION

DNA methylation introduces genetic variation in a conservative manner by altering the expression and function of DNA without permanently changing the sequence. Bacterial epigenetics, primarily in the form of DNA base methylation, has an outsized role in the regulation of various cellular processes (1 – 7). The methylated DNA bases found in bacteria and archaea include N^6^-methyladenine (6mA), 5-methylcytosine (5mC), and N^4^-methylcytosine (4mC), which are added by DNA methyltransferases either as part of a restriction-modification system or by solitary methyltransferases without associated restriction enzymes, called orphan methyltransferases (1, 4, 8 – 11). In gammaproteobacteria, 6mA modified by the orphan methyltransferase Dam at GATC motifs is the most abundant modified base in the genome (12 – 15). There are 19,120 of these palindromic GATC motifs in the *Escherichia coli* K-12 genome, or one GATC site on average every ~250 bp (Fig. S1) (16). 6mA influences various processes including, but not limited to, replication timing, transposon mobility, gene expression, virulence, and methyl-directed mismatch repair (MMR) (1, 6, 17 – 28). Many phenotypes regulated by 6mA involve phase variation, such as the pathogenic fimbriae *pap* in uropathogenic *E. coli* and *std* in *Salmonella enterica* serovar Typhimurium (29 – 31). In these phase-variable phenotypes, whether gene expression is on or off depends on the methylation status of multiple nearby regulator binding sites (32). Thus, the short-term stability but long-term mutability of DNA methylation facilitates these phase-variable phenotypes by allowing for stable regulation of loci that can rapidly switch with epigenetic changes (33).

The most widespread use of 6mA across the *E. coli* genome is in directing MMR. Methyl-directed MMR GATC sites are nearly all methylated on both strands, except for the brief period after chromosome replication before the daughter DNA strand is methylated by Dam (19, 34). When a mismatch is detected by MMR proteins (MutHLS), the nearby hemimethylated GATC sites are used to direct the MMR protein complex to remove the mismatched base from the daughter strand (19, 23, 35, 36). In *E. coli*, inactivation of any of the MutHLS proteins disrupts MMR and leads to a ~150-fold increased mutation rate (37, 38). Since MMR relies on the presence of hemimethylated GATC sites, alterations to the function of Dam in *E. coli* and other gammaproteobacteria also reduce the effectiveness of MMR (18, 22, 39).

Given the role of MMR in preventing replicative errors from becoming permanent mutations, we hypothesize that maintaining MMR imposes selective pressure on the *E. coli* genome to maintain GATC sites and associated 6mA modifications. While 6mA can have local gene expression effects when GATC sites coincide with regulatory elements and transcription factor binding sites, MMR is likely the most significant use of (and influence on) 6mA genome wide. Thus, we predict that in the absence of MMR, *E. coli* experimentally evolved for thousands of generations will exhibit increased variability in per-site 6mA methylation and a genome-wide decrease in 6mA due to a sustained accumulation of epimutations that do not incur the strongly deleterious fitness effect of causing MMR dysfunction. There has been limited examination of the dynamics of 6mA in evolving bacterial populations (40, 41). Thus, in this study, we sought to investigate how the presence and absence of MMR influence 6mA methylation in evolving bacterial populations over an extended time period. To accomplish this, we created a differential methylation-calling pipeline designed for bacterial 6mA data from third-generation sequencing: CoMMA (Comparison of Microbial Methylated Adenines). Third-generation sequencing platforms (such as PacBio’s SMRT sequencing or ONT’s Nanopore sequencing) allow for accurate and cost-effective identification of DNA base modifications. Using Nanopore sequencing and CoMMA, we first characterized genome-wide methylation frequency at GATC sites in *E. coli* MG1655. Then, focusing on clones isolated from evolved populations with initially WT and MMR-deficient genetic backgrounds, we characterized how 6mA methylation frequency changed among all 19,120 GATC motifs after 2,000 generations of experimental evolution in batch culture (42). We then compared our findings to mutation accumulation studies measuring spontaneous mutation rates in *E. coli* to determine how MMR constrains genetic and epigenetic evolution at GATC sites (37). Together, our results illustrate the evolvability of 6mA and its potential to contribute to heritable change in bacterial populations.

## RESULTS

### 6mA methylation status varies between genomic features in *E. coli* MG1655

**Fig 1:**
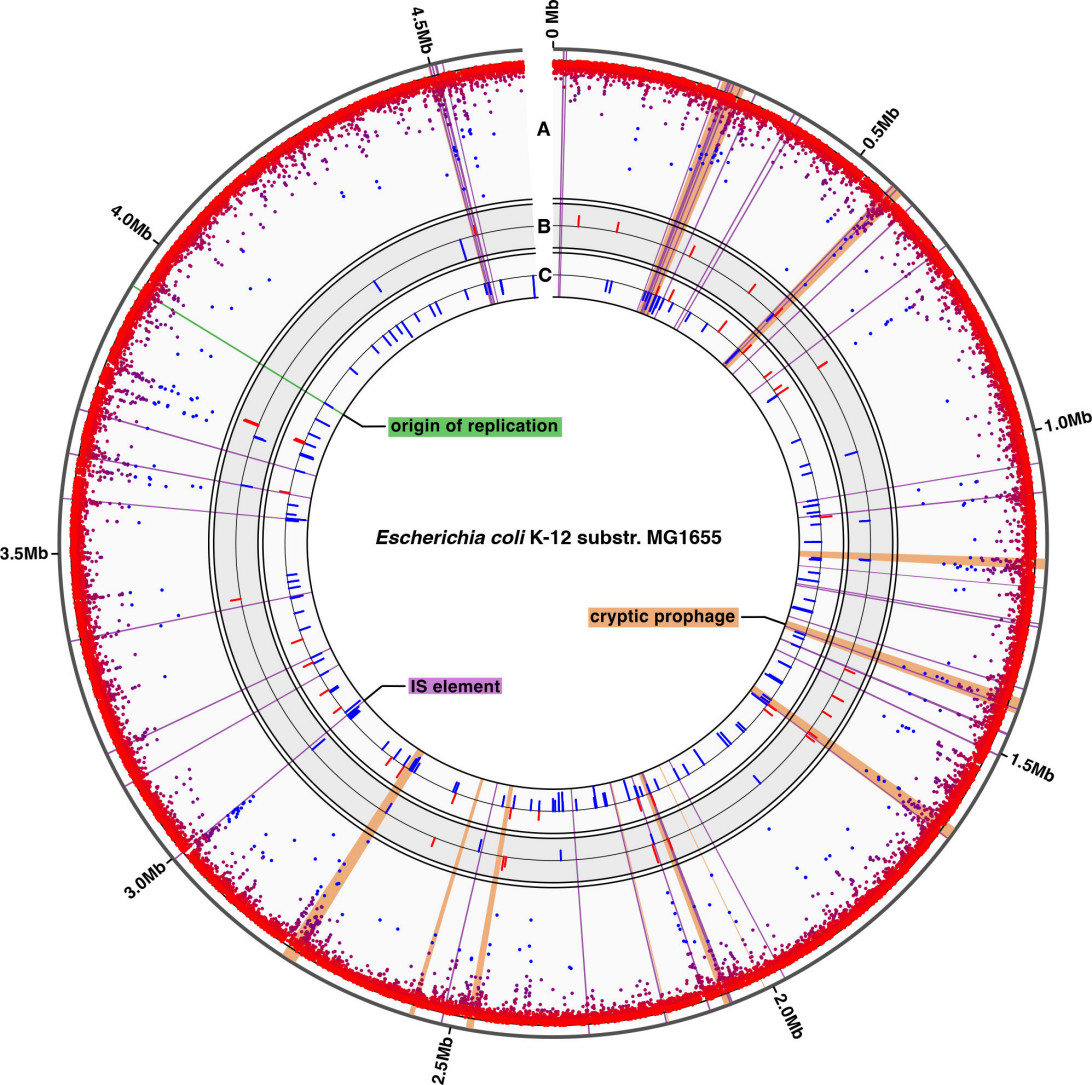
Genome-wide 6mA methylation in *E. coli* K-12 substr. MG1655. The origin of replication (green), IS elements (purple) and cryptic prophages (orange) are shown for reference. (**A**) Percent methylation at GATC sites in the ancestral WT MG1655 strain. Each point represents one GATC site and points are colored to reflect percent methylation from 0% (blue, inside) to 100% (red, outside). (**B and C**) Differentially methylated GATC sites in evolved WT clones (**B**) and MMR- (**C**) clones. Red bars are sites with increased methylation and blue bars are decreased methylation, and the height of the bar reflects the difference in percent methylation between the ancestor and the evolved clones. Each ring ranges from −25% to 25%.

We first measured genome-wide methylation in *E. coli* K-12 MG1655 (strain PFM2) to characterize methylation trends in our wild-type ancestor (37). Briefly, three colonies from an overnight culture were sequenced on an ONT MinION portable sequencer. Nanopore sequencing produced a median read depth of 57 reads per GATC site, and there was no significant strand bias in coverage (Fig. S2). To measure methylation from Nanopore reads, we used the methylation classification program Megalodon to calculate the proportion of methylated reads to total reads at each GATC site called the percent methylation (Data set S1) (43). Previous benchmarking studies have shown Megalodon to be one of the most reliable methylation callers when applied to data generated from Nanopore sequencing (44, 45). As there is currently no accepted gold standard methylation calling method, we also compared the performance of multiple independently developed methylation calling programs. With our data, Megalodon most consistently identified the same GATC sites as methylated or hypomethylated (<60% methylated) and correctly identified stably hypomethylated sites that were reported previously in studies that investigated methylation of select loci with biochemical methods (see Materials and Methods, Supplemental Text S1; Figs. S3 through S5; Table S1; Table S2) (34, 46 – 49). After classifying methylated sites with Megalodon, all GATC sites were annotated with data from RegulonDB, a curated database of *E. coli* K-12 transcriptional regulatory networks (50). Consistent with previous reports, most GATC sites were almost completely methylated, with a median genome-wide percent methylation of 97% (Fig. 1; Fig. S1) (8, 34, 40, 51). Methylation profiling revealed that 177 double stranded GATC sites are hypomethylated on at least one strand, of which 138 were hemimethylated and 39 were hypomethylated on both strands (Table S3). Intergenic GATC sites were significantly less methylated compared to those within coding sequences (median percent methylated reads of 93% compared to 97%; Wilcoxon rank sum test, *P* < 2.2 × 10^−16^). This decrease at intergenic sites is likely due to the higher proportion of transcriptional regulatory regions and transcription factor binding sites in intergenic regions, where these sites may exclude binding of the Dam methyltransferase to GATC sites.

**Fig 2:**
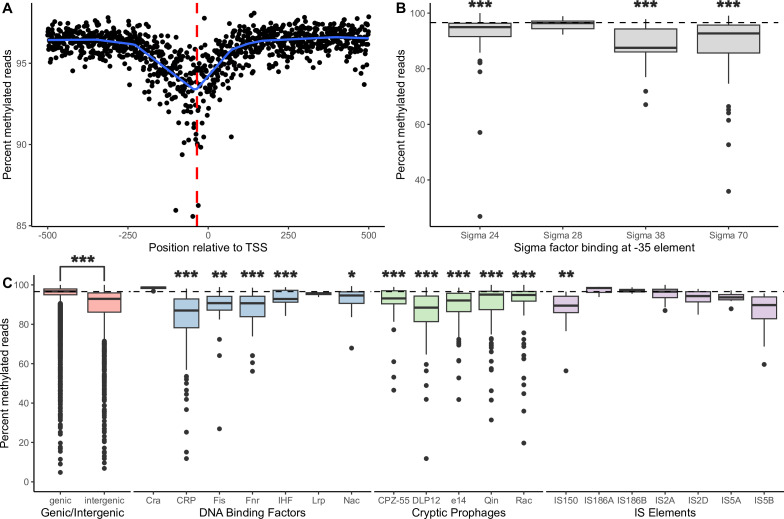
Genome-averaged methylation decreases around core promoter elements. (**A**) Median methylation at GATC sites aligned relative to the transcription start sites (TSS) for all promoters. Points represent the median methylation for all GATC sites at that position. The smoothed median was calculated by additive quantile regression smoothing (blue line). The −35 position relative to the TSS is shown for reference (dashed red line). (**B**) Comparison of percent methylated reads at −35 elements by specific sigma factor binding. Boxplots show the median, first quartile, and third quartile. Sigma factors were compared to the genome-wide median percent methylation (97%, dashed line) with a one-sample Wilcoxon signed-rank test with Benjamini-Hochberg correction. *P*-values are shown in B and C as ***<0.001, < **<0.01, < *<0.05. All groups without stars are not significant. (**C**) Different genomic features showed variable methylation status. Percent methylation of GATC sites within each feature type was compared to the genome-wide median percent methylation (97%, dashed line) with one-sample Wilcoxon signed-rank tests with Benjamini-Hochberg correction.

For a broader view of the factors that influence GATC methylation and to determine if hypomethylation was associated with GATC sites that may be shielded by protein binding, we looked at sites within intergenic regions where DNA-protein interactions likely occur, such as sigma factor binding sites and binding sites of other transcriptional regulatory proteins (2, 33, 48, 48, 52). Overall, within these intergenic regions, there is a general decrease in methylation around promoters. GATC sites within −10 and −35 promoter elements were significantly less methylated (95.2% for −10 elements, P_-10_ = 6.7 × 10^−8^; 90.4% for −35 elements, P_-35_ = 6.7 × 10^−10^, Wilcoxon signed-rank test with Benjamini-Hochberg correction) than the genome-wide median percent methylation (96.6%). This is best observed when methylation is averaged across all promoter regions in the genome; there is a lower frequency of methylated reads around 100 bp before and after the transcription start site, with a maximum decrease in methylation of ~5% centered on the −35 element (Fig. 2A; Fig. S6). Moreover, −35 promoter elements known to be bound by σ^70^, σ^38^, and σ^24^ had significantly lower percent methylation than the genome-wide median (Wilcoxon signed rank test with BH correction: P_sigma70_ = 2.0 × 10^−10^; P_sigma38_ = 7.0 × 10^−4^; P_sigma24_ = 1.6 × 10^−5^) (Fig. 2B).

In addition to core promoter elements, binding sites of other transcriptional regulatory proteins within intergenic regions also exhibited differences in their degree of hypomethylation (Fig. 2C; Fig. S7). Specifically, the global regulators CRP, Fis, Fnr, and Nac were significantly less methylated than the genome-wide median (Wilcoxon signed-rank test with BH correction: P_CRP_ = 2.0 × 10^−18^; P_Fis_ = 4.2 × 10^−3^; P_Fnr_ = 9.1 × 10^−6^; P_Nac_ = 4.98 × 10^−2^). Differences in methylation were also observed within coding regions for a few genomic features (Fig. 2C). Across the 12 cryptic prophages in the *E. coli* K-12 genome, significantly decreased methylation is observed in 9 of them: CP4-44, CP4-57, CP4-6, CPS-53, DLP12, e14, Qin, Rac, and PR-Y (CP4-44: 94.0% methylation, *P* = 6.2 × 10^−4^; CP4-57: 92.3%, *P* = 9.4 × 10^−16^; CP4-6: 95.3%, *P* = 2.5 × 10^−13^; CPS-53: 92.4%, *P* = 4.7 × 10^−6^; DLP12: 88.5%, *P* = 3.7 × 10^−13^; e14: 92.1%, *P* = 1.1 × 10^−8^; Qin: 95.1%, *P* = 2.9 × 10^−6^; Rac: 94.9%, *P* = 4.3 × 10^−8^; PR-Y: 93.8%, *P* = 2.6 × 10^−2^; Wilcoxon signed-rank test with BH correction) (53, 54). Of the various IS elements, *IS*150, *IS*4, *IS*30A, *IS*30C, and *IS*30D had significantly decreased methylation (*IS*150: 89.4% methylation, *P* = 2.6 × 10^−3^; *IS*4: 94.1%, *P* = 1.3 × 10^−2^; *IS*30A: 89.7%, *P* = 4.3 × 10^−2^; *IS*30C: 85.8%, *P* = 1.5 × 10^−2^; *IS*30D: 86.7%, *P* = 2.2 × 10^−2^) (55 – 57). Lastly, some genomic features, such as REP elements and terminator sequences, do not show measurable differences in 6mA methylation compared to the genome-wide average. However, these regions either do not contain many GATC sites or are poorly annotated. Comparing sequencing replicates from future studies across a variety of conditions, combined with improving genomic annotations, could allow for the detection of higher-resolution methylation differences and the characterization of methylation on abundant but unannotated sites.

### Differential methylation analysis shows that MMR- clones evolve decreased GATC methylation

After characterizing a baseline *E. coli* K-12 substr. MG1655 6mA methylome, we compared GATC methylation across the experimental ancestor PFM2 strain and eight isolated clones from populations that were experimentally evolved in batch culture for 2400 generations with either intact (WT) or disrupted mismatch repair (MMR-) (42). Briefly, CoMMA adapts the differential methylation package methylKit to identify GATC sites with significant differences in methylation status between bacterial samples using logistic regression (58). For a broad view of how methylation evolves, we focused on two populations from each of the WT or MMR- genetic backgrounds; from each of these populations, we selected two clones and ensured each clone belonged to a different evolved subpopulation. This sampling scheme would allow us to evaluate how methylation evolves within and among populations depending on their ability to conduct methyl-directed mismatch repair.

**Fig 3:**
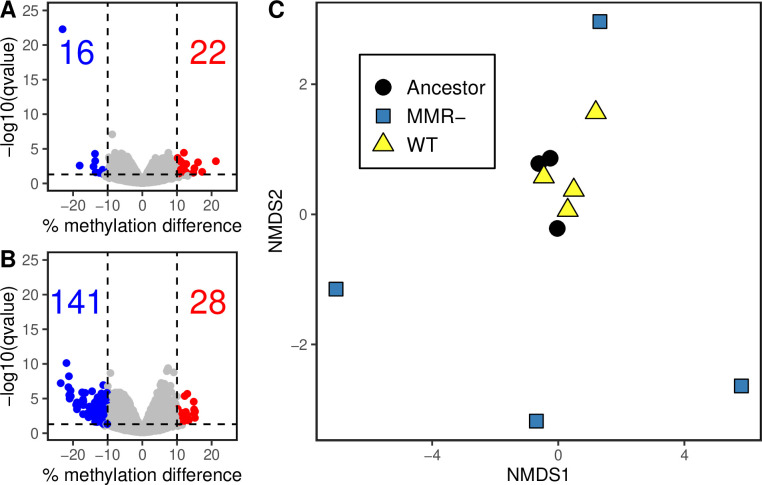
Differentially methylated GATC sites between the ancestor and experimentally evolved (**A**) WT clones or (**B**) MMR- clones. Differential methylation was called for WT and MMR- clones compared to the ancestor strain using methylKit. Differential methylation was determined for each site as a percent methylation difference compared to the ancestor greater than 10% and a q-value less than 0.05. Numbers shown in each plot field represent the number of differentially methylated sites that increased (red) or decreased (blue) in methylation in the evolved condition compared to the ancestor. (**C**) Comparison of methylation across all sites between the ancestor and experimentally evolved clones. Euclidean distance between clones was calculated using normalized β-values and ordination was performed with NMDS. Each point represents one clone.

As with the ancestor, experimentally evolved clones were Nanopore sequenced, and methylated sites were called using Megalodon (Data set S2). The median genome-wide methylation percentage of both genetic backgrounds decreased during batch culture evolution with MMR- clones exhibiting a significantly larger median decrease in methylation (−0.15%) compared to evolved WT clones (−0.03%; Wilcoxon rank-sum test, *P* < 2.2 × 10^−16^, Fig. S8). Next, we determined which specific GATC sites were differentially methylated between the evolved clones and the ancestor (Fig. 3A and B). Using cutoffs *P* < 0.05 and percent methylation change >10%, there were significantly more differentially methylated sites in the MMR- clones (28 sites with increased methylation, 141 sites with decreased methylation) compared to the evolved WT clones (22 increased, 16 decreased; Fisher’s exact test, *P* < 2.2 × 10^−16^).

Both evolved backgrounds also had a significant overrepresentation of differentially methylated sites within certain genomic regions, especially in sites where DNA-protein interactions occur. Across the genome, differentially methylated sites in both WT and MMR- evolved clones appear to coincide with regions of low methylation in the ancestor and with cryptic prophages and IS elements (Fig. 1B and C). Sites that were hemimethylated and hypomethylated in the MG1655 ancestor were enriched for differential methylation in the evolved clones (Fisher’s exact test, *P* < 2.2 × 10^−16^; Table S3). Differentially methylated sites in both evolved backgrounds were significantly over-enriched in transcription factor binding motifs and IS elements (Fisher’s exact test with FDR correction, *P* < 0.05, Table 1). Sites with increased methylation in both evolved backgrounds were also over-enriched within cryptic prophages, and sites with decreased methylation were over-enriched in Rho-independent terminators. The MMR- evolved clones also showed enrichment for under-methylated sites within cryptic prophages (*P* < 2.2 × 10^−16^) and within promoters, specifically σ^70^-binding sites in the −35 region (*P* < 2.2 × 10^−16^).

**TABLE 1:** Overrepresentation of differentially methylated adenines in protein-DNA interaction motifs^*a*^

		Evolved WT				Evolved MMR-			
Feature type	# GATC sites in ancestor	Increased methylation	*P*-value	Decreased methylation	*P*-value	Increased methylation	*P*-value	Decreased methylation	*P*-value
Transcriptional regulator binding sites	313	7	0.095	12^*c*^	< 0.001	11 **^*b*^**	0.014	58 **^*c*^**	< 0.001
Origin of replication	22	0	1.000	0	1.000	0	1.000	1	0.647
10 element	116	2	0.465	2	0.428	4	0.187	6	0.299
35 element	60	1	0.590	1	0.572	1	0.694	12 **^*c*^**	< 0.001
Sigma 24 binding sites	67	2	0.294	1	0.590	2	0.380	4	0.299
Sigma 28 binding sites	11	0	1.000	0	1.000	0	1.000	0	1.000
Sigma 32 binding sites	2	0	1.000	0	1.000	0	1.000	0	1.000
Sigma 38 binding sites	17	0	1.000	0	1.000	0	1.000	3^*c*^	0.043
Sigma 70 binding sites	86	1	0.694	2	0.313	3	0.254	12 **^*c*^**	< 0.001
Genes	35,595	312	1.000	275	1.000	442	1.000	952	1.000
Rho-dependent terminators	8	0	1.000	0	1.000	0	1.000	1	0.380
Rho-independent terminators	46	1	0.514	4 **^*c*^**	0.003	0	1.000	6 **^*c*^**	0.011
IS elements	283	10 **^*b*^**	0.002	10 **^*c*^**	0.001	11 **^*b*^**	0.007	18 **^*c*^**	0.014
Cryptic prophages	795	15 **^*b*^**	0.038	11	0.249	24 **^*b*^**	0.001	62 **^*c*^**	< 0.001
No feature	1,191	35 **^*b*^**	< 0.001	30 **^*c*^**	**<** 0.001	44 **^*b*^**	< 0.001	134 **^*c*^**	< 0.001

^*a*^ *P*-values were calculated by one-tailed Fisher's exact test followed by BH correction.

^*b*^ Significant number of sites with increased methylation.

^*c*^ Significant number of sites with decreased methylation.

**Fig 4:**
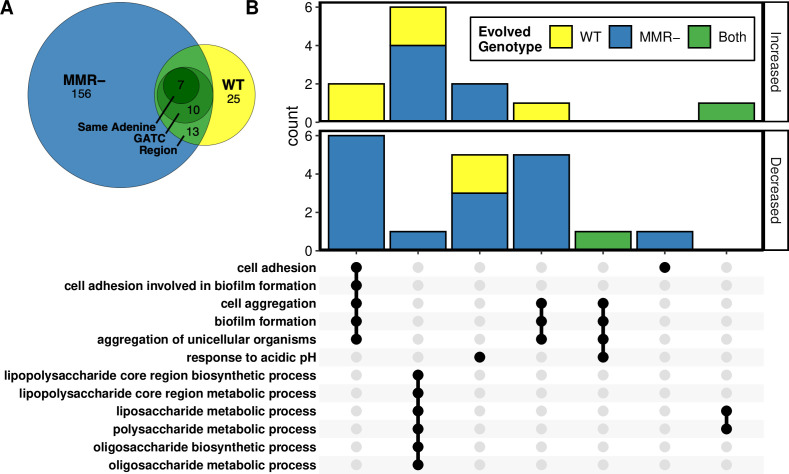
Gene ontology enrichment analysis of differentially methylated GATC sites. (**A**) Intersect between differentially methylated sites in evolved WT and MMR- genotypes. Sites in the same region are located in the same gene or the same intergenic region. (**B**) UpSet plot of significantly enriched gene ontology terms of genes associated with differentially methylated GATC sites that increased or decreased in methylation in evolved WT, MMR-, or both. Bar heights are the number of genes associated with the intersecting GO terms shown below.

Gene ontology (GO) enrichment analysis of genes containing or near differentially methylated sites revealed enrichment in genes associated with stress response, biofilm formation, and virulence factor production. Of the 194 differentially methylated sites identified between both WT and MMR- evolved clones, 13 sites were in the same gene or intergenic region between the two genotypes (Fig. 4A). Ten of these sites were in the same palindromic GATC site, and 7 of those were in the same adenine residue. GO enrichment analysis was then done on genes that contained differentially methylated GATC sites, or if the GATC site was not within a gene then the nearest gene was used. Genes that were less methylated in the MMR- clones were significantly enriched for biofilm formation and transcriptional regulators (Fig. 4B). Genes that were more methylated after evolution were enriched for biofilm formation in the evolved WT clones and for lipopolysaccharide metabolism in both WT and MMR- clones. Interestingly, 6 of the 15 biofilm genes identified belong to 3 different putative chaperone-usher fimbrial operons. These fimbriae are cryptic in *E. coli* K-12 under laboratory conditions, but when overexpressed they increase biofilm formation (59). Other biofilm genes *yfaL* and *ycgV* are homologs of the cryptic prophage-encoded autotransporter and adhesin Antigen 43 (Ag43 encoded by *flu*) (60). Both fimbriae and Ag43 are known to be subject to methylation-regulated phase variation in *E. coli* and related species (31, 60 – 62). Other enriched gene ontologies include lipopolysaccharide synthesis (including the non-functional O-antigen polymerase *wbbH*), acid stress response genes, phage defense genes, and various transcriptional regulators involved in stress response (Table S4) (63 – 66).

Comparison of the full 6mA methylomes between evolved clones and their ancestor revealed a non-deterministic change in 6mA in the absence of MMR consistent with random genetic drift. The percent of methylated reads at all GATC sites was compared between samples by visualizing using non-metric multidimensional scaling (NMDS; Fig. 3C). The evolved WT clones and the ancestor clones clustered together, showing that their 6mA methylomes remain similar after extended evolution (Pairwise PERMANOVA Ancestor vs. WT: P_Ancestor-WT_ = 0.168; PERMDISP2, ANOVA with Tukey’s HSD: P_Ancestor-WT_ = 0.99). In contrast, MMR- evolved clones diverge from the ancestor in random directions, and the MMR- clones do not cluster together. The centroid of the MMR- clones was near that of the ancestor and WT clones but significantly different than the WT clones, and MMR- clone dispersions were significantly greater than both the ancestor and WT groups (Pairwise PERMANOVA Ancestor vs MMR-: P_Ancestor-MMR_ = 0.195, WT vs MMR-: P_WT-MMR_ = 0.026; PERMDISP2, ANOVA with Tukey’s HSD: P_Ancestor-MMR-_ = 0.018, P_WT-MMR-_ = 0.010). This suggests that 6mA differences that occur during evolution in MMR- lines are non-deterministic, resulting in significant changes to methylation for each sample but not strongly in any one direction for the whole group. MMR- clones from the same population did not cluster together, indicating that shared evolutionary history does not have a large effect on broad 6mA differences over this time scale. Together, the significant number of under-methylated sites and the random divergence of differentially methylated sites in the MMR- clones compared to the ancestor suggest that 6mA is shaped by genetic drift in the absence of MMR.

### MMR- evolved lines accumulate GATC mutations at a rate that indicates relaxed selection

After identifying decreased maintenance of 6mA in the absence of MMR, we sought to genomically identify if the evolution of GATC sites in MMR- clones exhibited patterns of decreased evolutionary constraint. Here, we characterized the fraction of mutations that altered GATC sites during batch culture evolution across clones from both genetic backgrounds. After ~2,400 generations, MMR- clones had acquired a total of 959 mutations with 34 mutations (3.5% of mutations) causing a gain or loss of a GATC site (Table 2; Table S4). In contrast, the WT clones accumulated a total of 33 mutations, none of which had affected GATC sites. Since the relative lack of mutations in WT clones limited our statistical power, we compared the mutation rates of the clones that were evolved in batch culture to mutation rates calculated in a mutation accumulation (MA) experiment by Lee et al. (37). As MA experiments are conducted using culture conditions that minimize the effects of selection, comparing the GATC mutation rates of clones evolved in batch culture to the GATC mutation rates of MA lines allows us to determine how batch culture GATC mutation rates compare to the neutral expectation. Moreover, as Lee et al. (37) measured mutation rates by conducting MA with the same WT and MMR- PFM2 strain, these mutation rates directly reflect the expected rate of GATC mutation for the WT and MMR- clones if their GATC tetranucleotides are evolving under relaxed selection.

**TABLE 2:** Number of mutations in GATC sites in evolved clones

Background	Population*^a^*	Clones^*b*^	Mutations	GATC gain/loss	Percent mutations in GATC sites (this study)		Percent mutations in GATC sites (37)	
MMR	113		257	13	5.1			
		113–1	121	3	2.5			
		113–2	96	3	3.1			
	126		46	1	2.2			
		126–2	313	11	3.5			
		126–4	126	3	2.4			
	Total		959	34	3.5	(2.8–4.0)^*c*^	3.5	(2.8–4.3)^*c*^
WT	125		4	0	0			
		125–6	1	0	0			
		125–7	7	0	0			
	129		4	0	0			
		129–1	8	0	0			
		129–3	9	0	0			
	Total		33	0	0	(0.0–0.0)^*c*^	4.3	(2.5–6.7)^*c*^

^*a*^ Mutations shared between clones and occurred before subpopulation divergence.

^*b*^ Mutations unique to clones and occurred after subpopulation divergence.

^*c*^ Bootstrap 95% confidence intervals.

Reanalysis of the MA data to calculate mutation rates in tetranucleotide contexts revealed similar spontaneous GATC mutation rates for WT and MMR- MA lines (Table 2). As such, GATC sites should evolve at the same rate for both genetic backgrounds during batch culture evolution unless selection pressures on GATC sites are different based on the presence or absence of MMR. Notably, during MA, 3.5% of mutations in MMR- lines affect GATC sites. This percentage is equal to the fraction of mutations affecting GATC sites in the MMR- clones and is a stark contrast to the absence of GATC mutations in WT clones during evolution in batch culture. This similarity in the GATC mutation rates from the batch culture evolution and MA experiments suggests that batch culture MMR- clones acquire mutations in GATC sites at the neutral rate and that in the absence of functional MMR, GATC sites may be evolving under relaxed selection. Additional evidence that GATC sites and associated adenine methylation may be evolving under relaxed selection in MMR- clones include that the clones isolated from the MMR- population 113 both acquired mutations resulting in a non-synonymous D20A substitution proximal to the GATC recognition domain of *dam* methyltransferase (67) during evolution in batch culture. While the exact consequences of this mutation have not been examined, this substitution could affect DNA binding and GATC recognition, indicating that perfect Dam function may not be necessary in the absence of MMR. Together, these data suggest that MMR is a major factor in maintaining GATC sites and *dam* methyltransferase, and in the absence of MMR these loci are vulnerable to mutation.

## DISCUSSION

In this study, we present what is to our knowledge a first-of-its-kind high-resolution characterization of the *E. coli* K-12 MG1655 6mA methylome that includes a functional and genic classification of 6mA methylation. We identified patterns of reduced methylation at intergenic regions, promoter elements, DNA binding sites, and cryptic prophages. This reduced methylation could result from the physical exclusion of Dam methyltransferase at these sites and potentially cause downstream effects on gene regulation and stress response. The ability to acquire high-resolution methylation data opens the door for future investigations on the specific effects of these methylation differences when paired with transcriptomic and biochemical experiments.

We also created and applied the CoMMA pipeline to investigate the effect of 2,400 generations of evolution on WT and MMR- *E. coli* MG1655 clones. We found that the MMR- clones exhibited a significant decrease in methylation, indicating that 6mA is somewhat dispensable in the absence of MMR. While methylation was largely stable at the majority of GATC sites, MMR- clones exhibited significantly more differentially methylated sites than WT clones. Interestingly, these differentially methylated sites are often located in DNA binding sites. Adenine modification can affect specific binding affinity where 6mA coincides with a DNA binding site (68, 69). Without MMR imposing a genome-wide pressure to maintain 6mA for the purpose of directing DNA repair machinery, MMR- clones are free to alter 6mA at these sites with a bias toward decreased methylation, likely due to binding competition between transcription factors and the methyltransferase. Altered 6mA methylation that affects transcription factor binding can result in downstream effects and further alter cellular phenotypes. Our study demonstrates that differential methylomics is a powerful approach that can directly resolve differences between clones that are not apparent from genetic changes alone. Thus, the CoMMA pipeline is a useful tool that can complement genetic approaches.

An interesting observation from our study involves one of the MMR- evolved populations (P113), which acquired a mutation in the *dam* adenine methyltransferase resulting in a single amino acid substitution (DamD20A). The consequence of this mutation is unclear, although this residue is proximal to three S-adenosylmethionine-binding sites at K10, K14, and D54, and could potentially affect protein folding (67). While there are no broad methylation differences within these clones compared to others of the same background, a DamD20A substitution may have contributed to the observed changes in methylation in these clones. Further studies are needed to investigate if this mutation altered Dam activity and how this affects genome-wide methylation patterns.

One potential impact of our findings is how 6mA methylation may influence the mutational spectrum and adaptive landscape of bacteria. DNA base modifications can affect the rates of spontaneous deamination; for instance, 5mC is vulnerable to deamination and contributes to the C→T mutation rate (70 – 72). Similarly, adenine and 6mA may deaminate to hypoxanthine, which pairs with cytosine during replication (73, 74). While the spontaneous deamination rates of adenine versus 6mA have not been exhaustively studied, recent work identified a positive correlation between 6mA and A→G transitions in *Neisseria meningitidis* (75). Although we did not detect any immediate differences in the mutation spectrum of WT and MMR- MA lines at GATC sites, genome-wide decreases in 6mA after MMR loss may eventually result in a decreased A→G transition rate. This change to the mutational spectrum could potentially affect how MMR- or Dam- cells adapt to changing environments and may influence the trajectory of their evolutionary paths.

This study is the first-ever analysis of differential methylation between laboratory evolved bacterial lineages at this time scale. The ability to identify changes to 6mA at GATC sites over evolutionary time creates opportunities for the seamless integration of genetic and epigenetic data. Nanopore sequencing and pipelines like CoMMA are sufficiently reliable, affordable, and high-throughput to powerfully analyze genetic and epigenetic evolution in bacteria. However, many factors can influence methylation studies, including sequencing chemistry, the methylation calling program, the biological conditions of the sample, and bioinformatic filtering, data processing, and statistical analysis; thus, care must be taken to identify and account for these systematic biases in comparative studies. The development of standardized tools for methylation analyses like CoMMA is a step toward addressing some of these biases. As more studies uncover how bacterial DNA methylation affects gene expression, differential epigenetic analyses can be extended through time or between bacterial isolates and help to elucidate the relationship between mutation, epimutation, and gene expression as it relates to adaptation to challenging environments (40, 41, 76, 77).

## MATERIALS AND METHODS

### Bacterial strains and culture conditions

All bacterial strains were cultured and sequenced to identify mutations as part of a long-term batch culture experimental evolution study that has been described previously (42). Briefly, the WT ancestor strain PFM2 is a prototrophic derivative of *E. coli* K-12 MG1655, and the MMR-deficient strain PFM5 is directly derived from PFM2 by introducing an in-frame deletion of the *mutL* gene (37, 42). Experimental evolution in batch culture was started by inoculating 10 mL of LB-Miller broth (BD Difco) in 16 × 100 mM glass culture tubes with a single colony of the progenitor strain (42). Cultures were maintained shaking at 175 rpm, alternating transfers after 24 hours of growth at 37°C and 48 hours of growth at 25°C. Cultures of interest in this study were transferred by thoroughly vortexing the culture tube and inoculating 9 mL of LB-Miller broth with 1 mL of culture, resulting in a transfer of about 10^9^ cells (42). Populations were grown and transferred for 2,000 total generations of growth (2.5 years). After 2,000 generations, samples from the evolving populations were streaked for isolation and eight random clones were saved in 40% glycerol at −80°C for further and future experimentation.

### Nucleic acid isolation and Nanopore sequencing

Evolved and WT ancestor clones were revived from frozen storage on LB agar by incubating overnight at 37°C. For each clone, a single isolated colony was randomly selected and restreaked on to fresh LB agar and again incubated overnight at 37°C for DNA extraction. Following overnight incubation, colonies were collected from the agar using a 10 mL loop and inoculated directly into DNA extraction buffer, and DNA was further isolated with the UltraClean Microbial DNA Kit (Qiagen). Following recommendations from ONT, extracted DNA was assessed for quantity using the Qubit High Sensitivity dsDNA kit and quality using the Genomic DNA ScreenTape on a TapeStation 4200 (Agilent). Libraries were then prepared for Nanopore sequencing using the PCR-free Rapid DNA Sequencing Kit (SQK-RAD004, Nanopore) paired with the Rapid Barcoding Kit (SQK-RBK004). Prepared libraries were pooled and loaded on to a R.9.4.1 flow cell and sequenced on a MinION for 48 hours with live basecalling in MinKNOW deactivated.

### Bioinformatics and sequencing analysis

To determine the precision of methylation calling, we compared results for each of four methylation calling pipelines across three Nanopore sequencing replicates of the WT ancestor PFM2 and determined the number of inconsistently called sites. Initial Nanopore basecalling by converting fast5 files into fastq was conducted using Guppy GPU (v. 3.1.5+781ed57). Following basecalling, samples were demultiplexed with Guppy GPU. Average read length across all samples is 5,583 ± 275 nucleotides (± s.e.m). Following basecalling and demultiplexing, we evaluated four different pipelines [Tombo (v.1.5), mCaller (v.0.3), Deepsignal (v.0.1.6), and Megalodon (v.0.1.0)] for their precision and accuracy to identify base modifications and determine methylated adenines within GATC sites (43, 46, 47, 78). Both Tombo and Megalodon were created by ONT for methylation calling, with Tombo being an older release that relies on statistical methods to annotate non-canonical bases and Megalodon being a newer release based on machine learning with deep neural networks (43, 47). To increase accuracy, Megalodon can be run with a VCF file containing the position and identity of known variants, for which we used Illumina sequencing data for evolved clones that was previously published (42). For pipelines not developed by ONT: DeepSignal applies a deep neural network to data that has been preprocessed by Tombo; and mCaller applies a deep neural network to data that has been preprocessed by Nanopolish. When running the mCaller pipeline we used Nanopolish v.0.11.1). Across all samples, the mean read coverage across GATC sites was 57 reads per site (Fig. S2).

Identification of mutations affecting GATC sites in evolved clones was conducted using previously published WGS Illumina sequencing data. To identify the rate of mutations affecting GATC sites under minimal selection, we utilized previously published mutation accumulation data for the *E. coli* strains that served as the experimental ancestor for the WT (PFM2) and MMR- (PFM5, PFM2:∆*mutL*) (37). For both the experimentally evolved clones and the MA lines, mutations were called using GATK best practices and the genic location of mutations was annotated using SnpEff v.4.3 by comparing VCF files of identified mutations for each clone to the *Escherichia coli* K-12 str. MG1655 reference genome (INSDC accession: U00096.3) (79). To confirm a mutation occurred in a GATC site, the tetranucleotide context of each mutation was identified with the fill-fs -l 5 function from vcftools (v.0.1.12) to annotate each variant with the flanking five upstream and downstream nucleotides (80).

GATC sites were annotated as within different genomic features using all combined databases in RegulonDB v.10.9 (81). These annotations were concatenated into a BED file, which was used to annotate GATC sites with the CoMMA function annotateMethylSites.

### Differential methylation calling

Differential methylation analysis was performed in R using CoMMA, a package that serves as a wrapper for methylKit v.3.13 (58). GATC sites that had acquired mutations in any clone were removed from differential methylation analysis so that only GATC sites shared between samples were considered. To ensure the reliability of differential methylation analysis, CoMMA includes the following modifications to the standard methylKit workflow: (i) any sites covered by fewer than 10 reads were excluded from analysis; (ii) methylation coverage at each site was normalized by median coverage; and (iii) sites were ultimately considered to be differentially methylated based on a q-value cutoff of <0.05 and percent methylation difference >10%. Differential methylation was called separately between the ancestor and either the evolved MMR- or WT clones. Differential methylation was calculated by logistic regression with a sliding linear model (SLIM) to correct for multiple hypothesis testing (58, 82).

### Statistical analysis and ordination

Statistical analysis comparing percent methylation (or ꞵ-value) was performed on M-values to remove the heteroscedasticity observed in ꞵ-value distributions (83). M-values were calculated as shown in Equation 1:


(1) $$M\;=\;log2\left(\frac{\beta }{1\;–\;\beta \;+\;0.001}\right)$$

All statistical analysis was done in R. M-values of GATC sites within different genomic features were compared using the Kruskal-Wallis rank sum test, and each feature was compared to the genome-wide median M-value using a one-sample Wilcoxon signed-rank test with Benjamini-Hochberg correction for multiple comparisons (84).

Ordination of samples was performed after normalizing ꞵ-values. Since ꞵ-values are dependent on coverage, samples with low coverage stand out when visualized with non-metric ordination methods. Therefore, before ordination, we normalized coverage and methylated read counts between samples to reduce the effects of coverage on ordination distance. First, the coverage of all samples was normalized using quantile normalization (85). Then, a scaling equation was calculated for each sample using the simple linear regression line between coverage and normalized coverage. This scaling equation was then applied to each sample’s coverage and methylated read count, both of which were used to calculate normalized ꞵ-values. Ordination of samples was performed using the R package vegan v.2.6–4 (86). Non-metric multidimensional scaling was used to visualize sample clustering based on a Euclidean distance matrix calculated from normalized ꞵ-values. PERMANOVA and PERMDISP2 were done with vegan functions adonis2 and betadisper, respectively.

Gene ontology enrichment analysis was performed using R package clusterProfiler v.4.6.2 and the Bioconductor *E. coli* K-12 annotation v.3.8.2 (87 – 89). Genes enriched for biological process ontologies were filtered by a q-value cutoff of 0.05, and redundant GO terms were removed using the clusterProfiler “simplify” method.

## Data Availability

Newly generated sequencing data for the identification of nucleotide modifications can be downloaded from SRA, BioProject PRJNA912686. Code and reformatted data for reproducing this analysis are found at https://github.com/BehringerLab/Methylation. The CoMMA R package is available at https://github.com/carl-stone/CoMMA.

## SUPPLEMENTAL MATERIAL

Data Set S1 mbio.01289-23-s0001.xlsx Methylation data for the three ancestor replicates called with all 4 methylation calling software packages.

Data Set S2 mbio.01289-23-s0002.xlsx Methylation data for the Ancestor and Evolved clones called with Megalodon.

Text S1 mbio.01289-23-s0003.pdf Evaluation of methylation calling software packages.

Supplemental Figures mbio.01289-23-s0004.pdf Figures S1 to S8.

Table S1 mbio.01289-23-s0005.xlsx Classification of methylation status at double-stranded GATC sites for each methylation calling program.

Table S2 mbio.01289-23-s0006.xlsx Proportion of methylated reads at nonmethylated GATC sites across all callers.

Table S3 mbio.01289-23-s0007.xlsx All hemimethylated and hypomethylated GATC sites in the WT K-12 MG1655 genome.

Table S4 mbio.01289-23-s0008.xlsx GATC sites lost or gained by mutation in MMR- evolved clones.

ASM does not own the copyrights to Supplemental Material that may be linked to, or accessed through, an article. The authors have granted ASM a non-exclusive, world-wide license to publish the Supplemental Material files. Please contact the corresponding author directly for reuse.
