# Supplementary material for: Differential adenine methylation analysis reveals increased variability in 6mA in the absence of methyl-directed mismatch repair
Source: mBio. 2023 Oct 5;14(5):e01289-23. doi: 10.1128/mbio.01289-23 (PMC10653831; doi:10.1128/mbio.01289-23)
Supplement: Supplemental Figures — Figures S1 to S8. [file mbio.01289-23-s0004.pdf]

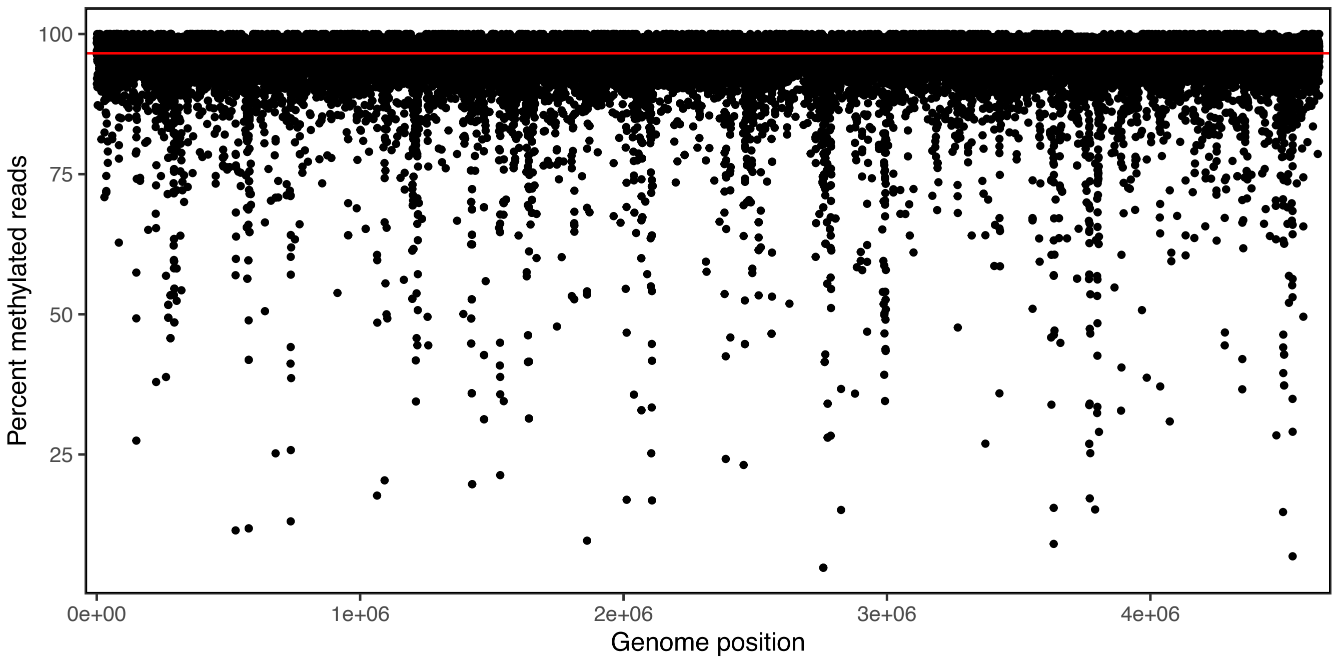

Figure S1. 6mA methylation across the MG1655 genome. Each point represents the mean percent methylation of three sequencing replicates of the K-12 substr. MG1655 ancestor strain at one GATC site. The genome-wide median methylation of 96.6% is shown for reference (red line).

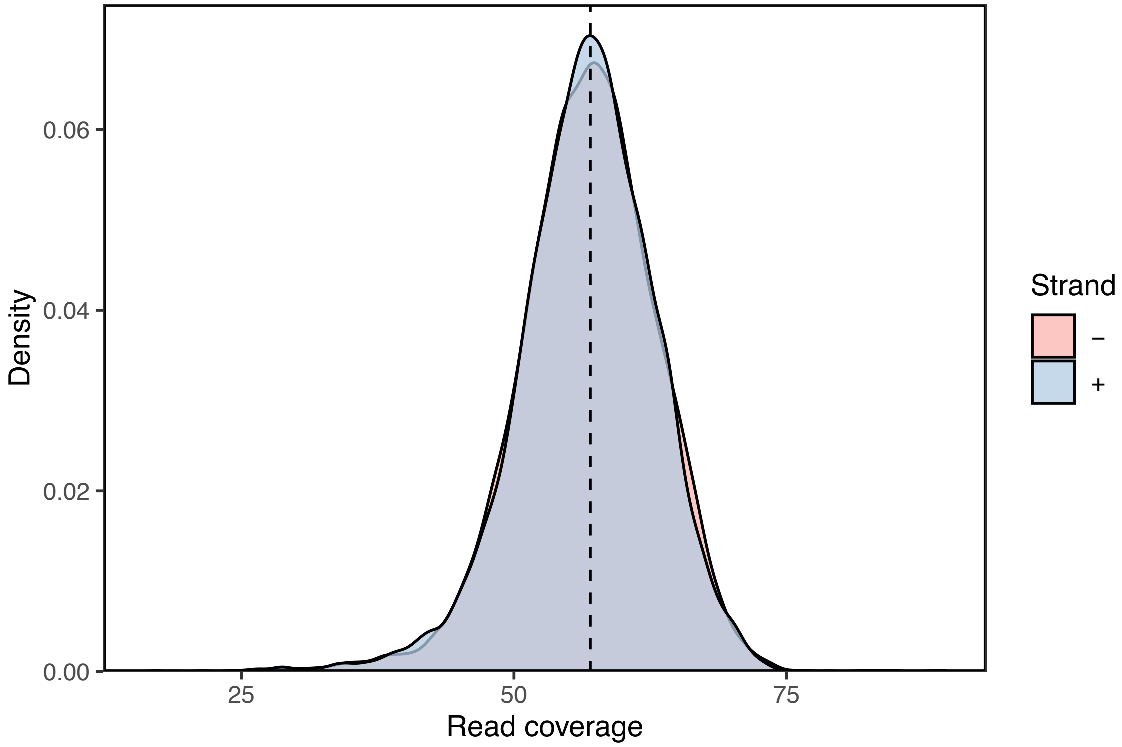

Figure S2. Average sequencing coverage of GATC sites by Nanopore sequencing. Coverage distributions for reads spanning GATC sites on the (+) and (-) strands are shown separately. Distributions have the same median of 57 reads (dashed line).

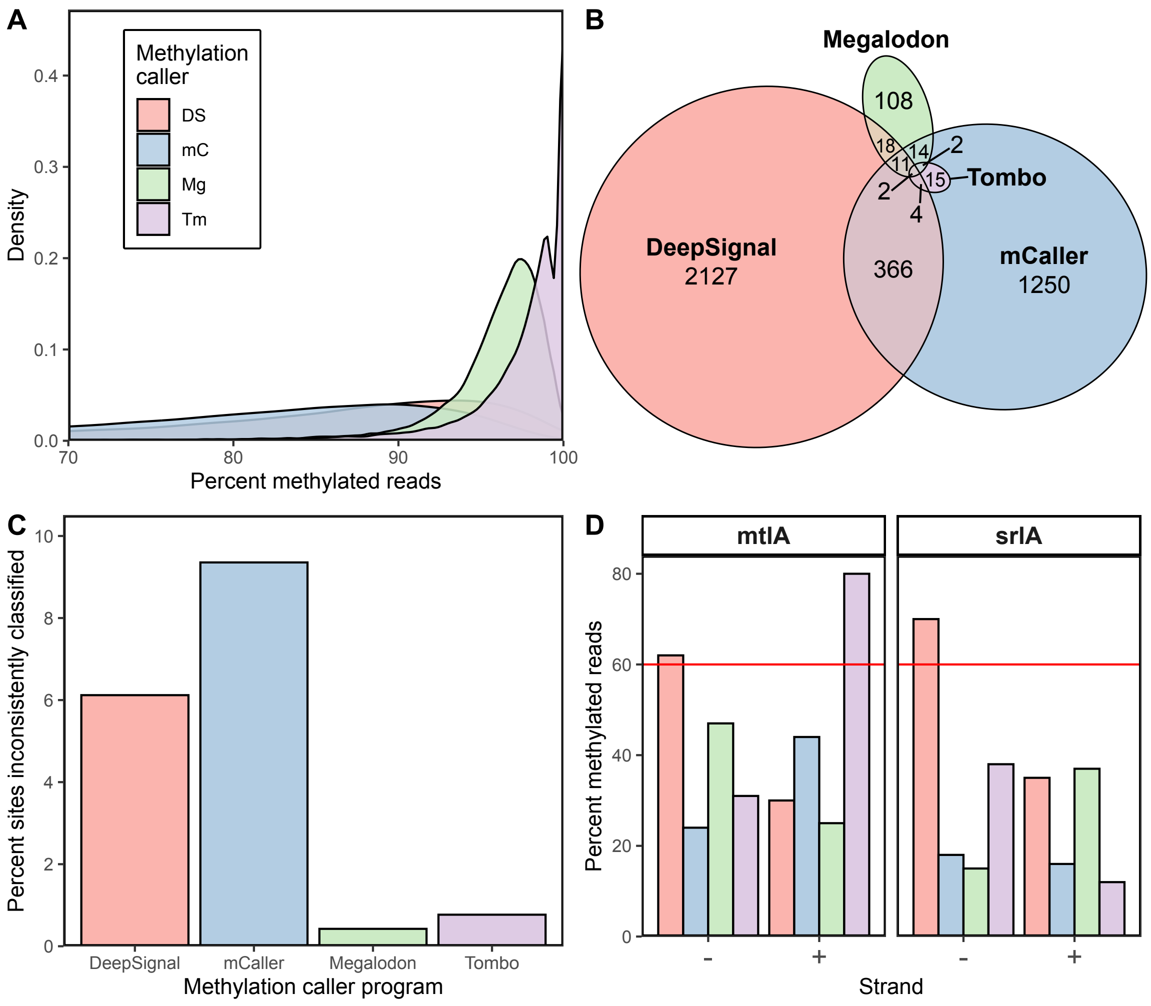

Figure S3. Comparison of each methylation calling program's performance when identifying methylated reads at GATC sites. (A) Distribution of percent of reads classified as methylated at all GATC sites in the *E. coli* K-12 substr. MG1655 genome for each methylation caller assessed in this study. Dashed lines represent median percent methylation across all GATC sites for, from left to right, mCaller (83%), DeepSignal (87%), Megalodon (97%), and Tombo (99%). (B) Overlap between GATC sites classified as hypomethylated across all three ancestral PFM2 *E. coli* K-12 substr. MG1655 replicates by each methylation calling program. Overlapping circles are drawn to scale relative to the number of sites represented. Each area is labeled with the number of shared GATC sites. (C) Percentage of methylated GATC sites inconsistently classified as methylated across the three ancestral PFM2 *E. coli* K-12 substr. MG1655 replicates for each program. (D) Average percentage of reads classified as methylated by each program at GATC sites previously reported to be nonmethylated in earlier studies though biochemical methods (mtlA: nt 3,771,971 / 3,771,972; srlA: nt 2,825,746 / 2,825,747) (48).

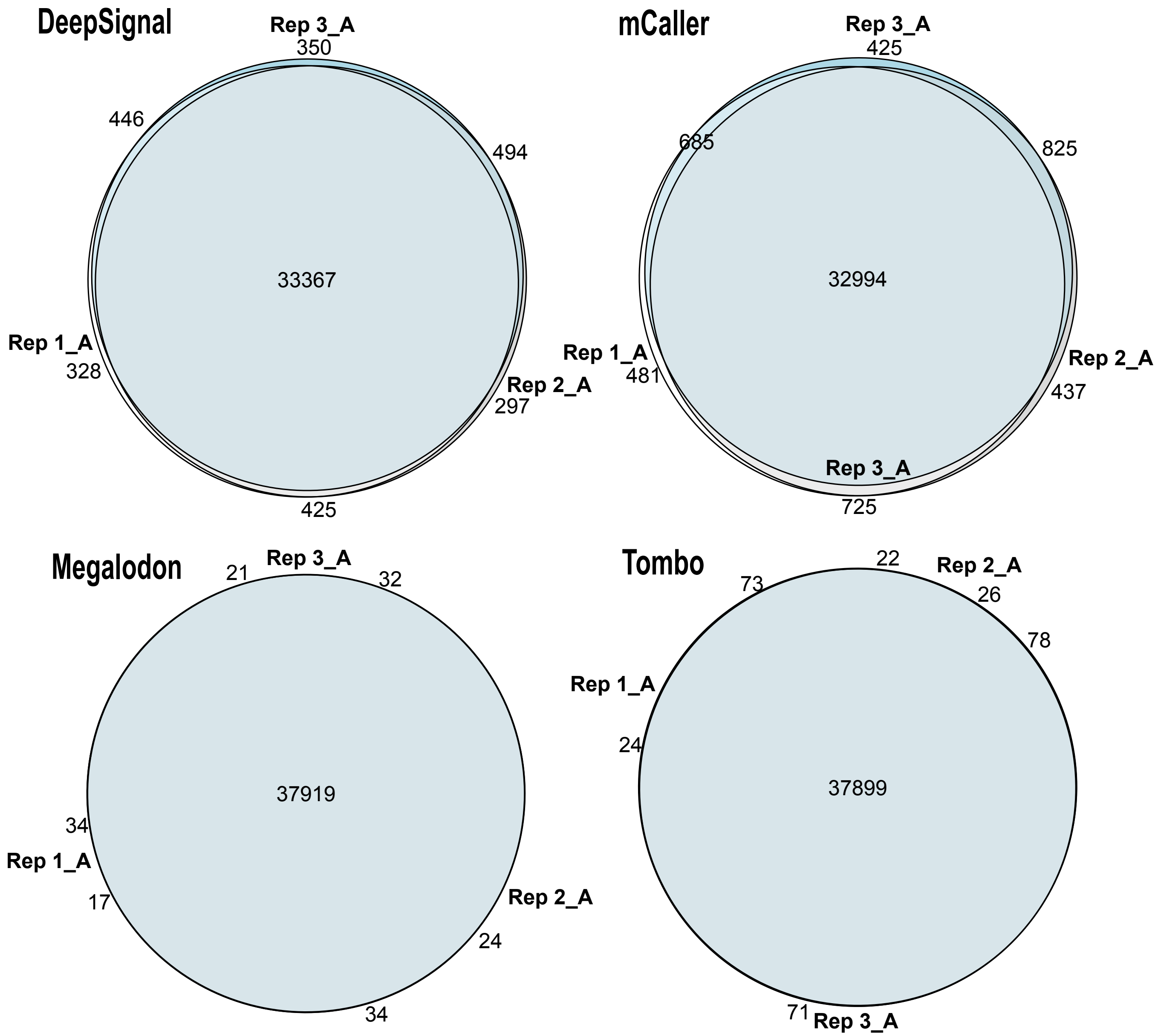

Figure S4. Overlap in sites identified as methylated between three sequencing replicates of *E. coli* K-12 substr. MG1655 (PFM2) for each caller. Venn diagrams describe the agreement across all three ancestor replicates for each methylation caller when identifying GATC sites as ≥60% methylated. Overlapping circles are drawn to scale relative to the number of sites represented. As *E. coli* genomes are expected to be highly methylated at the vast majority of GATC sites, this is reflected in the large mutual intersection of the Venn diagrams. For all callers the replicates are denoted by different colors: Ancestor replicate 1 (Rep 1_A; white), Ancestor replicate 2 (Rep 2_A; gray), Ancestor replicate 3 (Rep 3_A; blue).

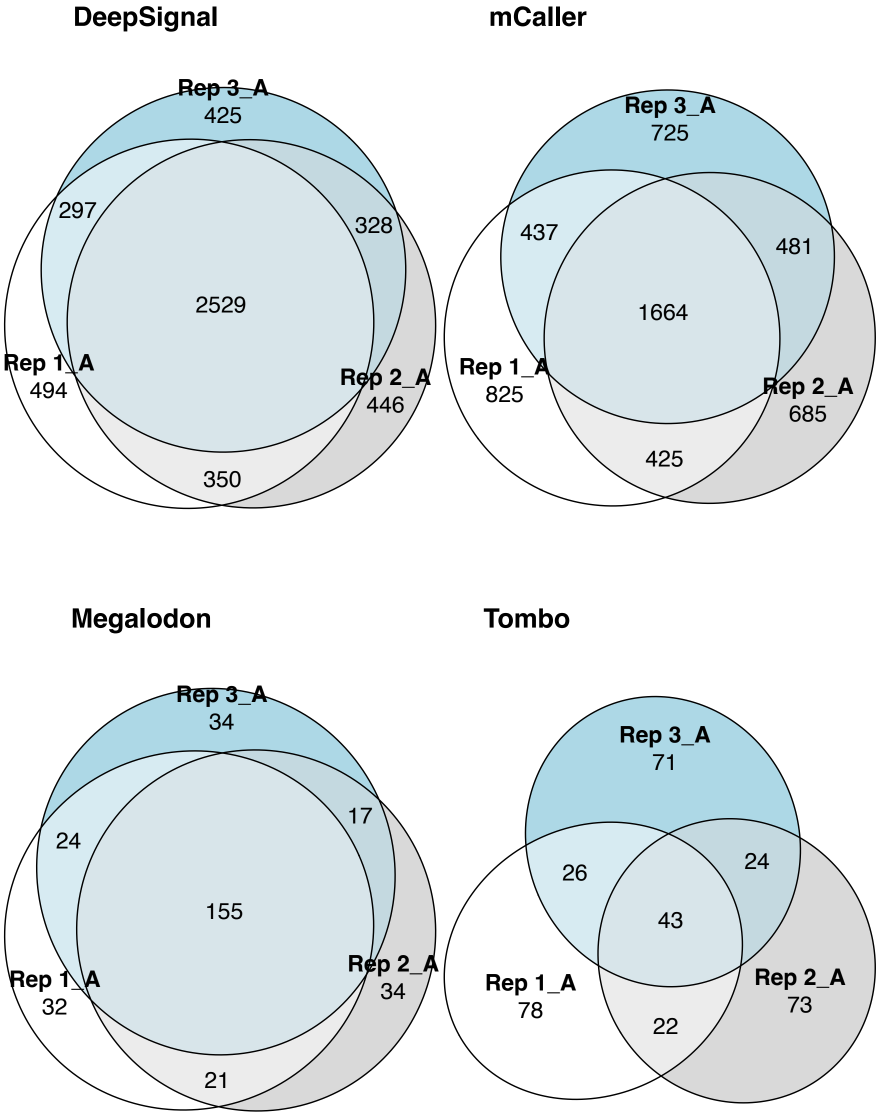

Figure S5. Overlap in sites identified as hypomethylated between three sequencing replicates of *E. coli* K-12 substr. MG1655 (PFM2) for each caller. Venn diagrams describe the agreement across all three ancestor replicates for each methylation caller when identifying GATC sites as <60% methylated. Overlapping circles are drawn to scale relative to the number of sites represented for each methylation caller. As hypomethylated GATC sites are expected to account for a small percentage of GATC sites across *the E. coli* genome, comparing agreement across hypomethylated sites provides a greater insight into the consistency of the different methylation callers. For all callers the replicates are denoted by different colors: Ancestor replicate 1 (Rep 1_A; white), Ancestor replicate 2 (Rep 2_A; gray), Ancestor replicate 3 (Rep 3_A; blue).

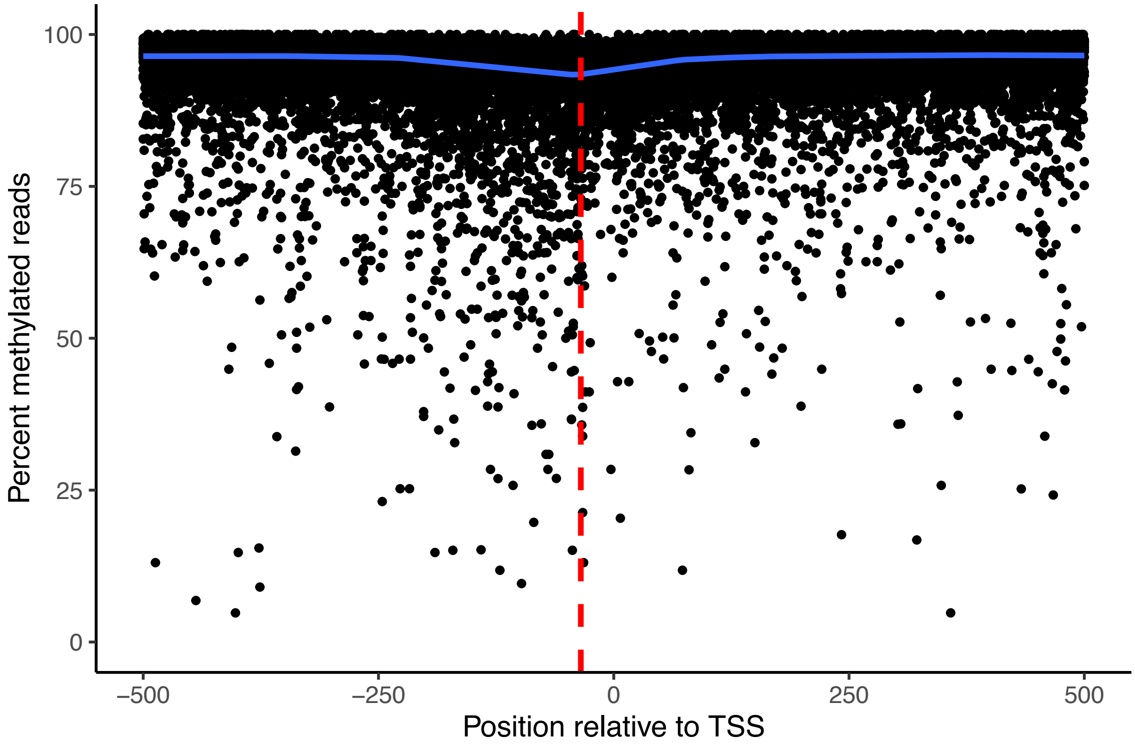

Figure S6. Genome-average methylation decreases around the transcription start site (TSS). Methylation at GATC sites aligned relative to the TSS for all promoters. Each point represents a single GATC site. The smoothed median was calculated by additive quantile regression smoothing (blue line), and the -35 position is shown for reference (dashed red line).

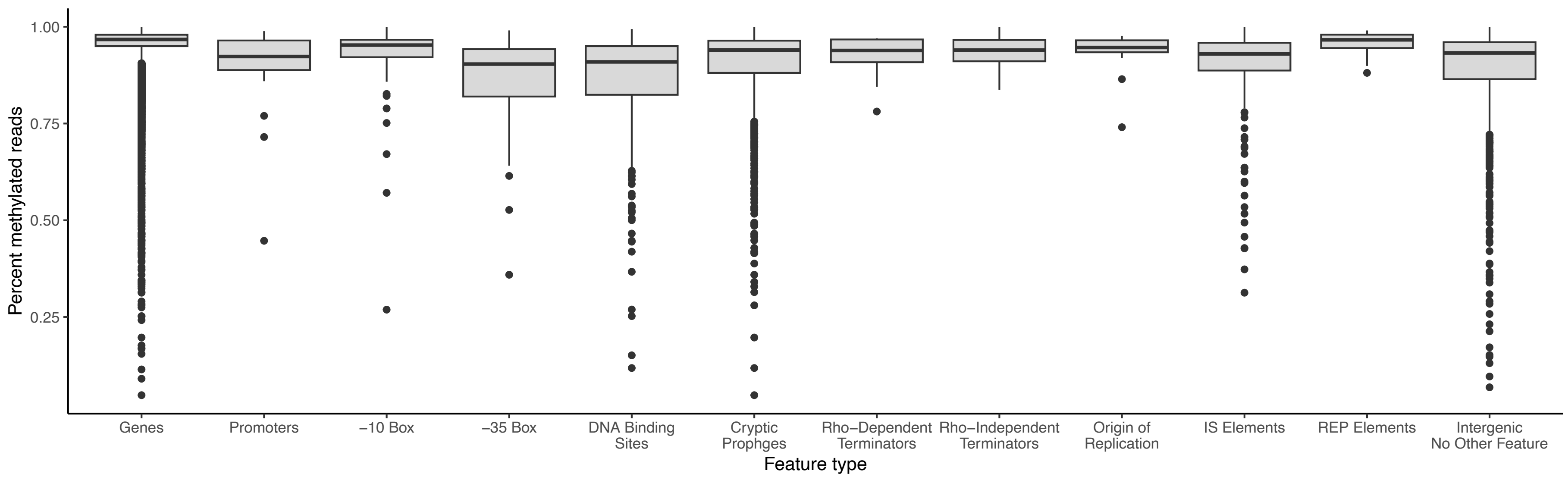

Figure S7. Average methylation at various genomic features in the K-12 MG1655 ancestor clone. Boxplots show the median, first quartile, and third quartile of the percent methylated reads.

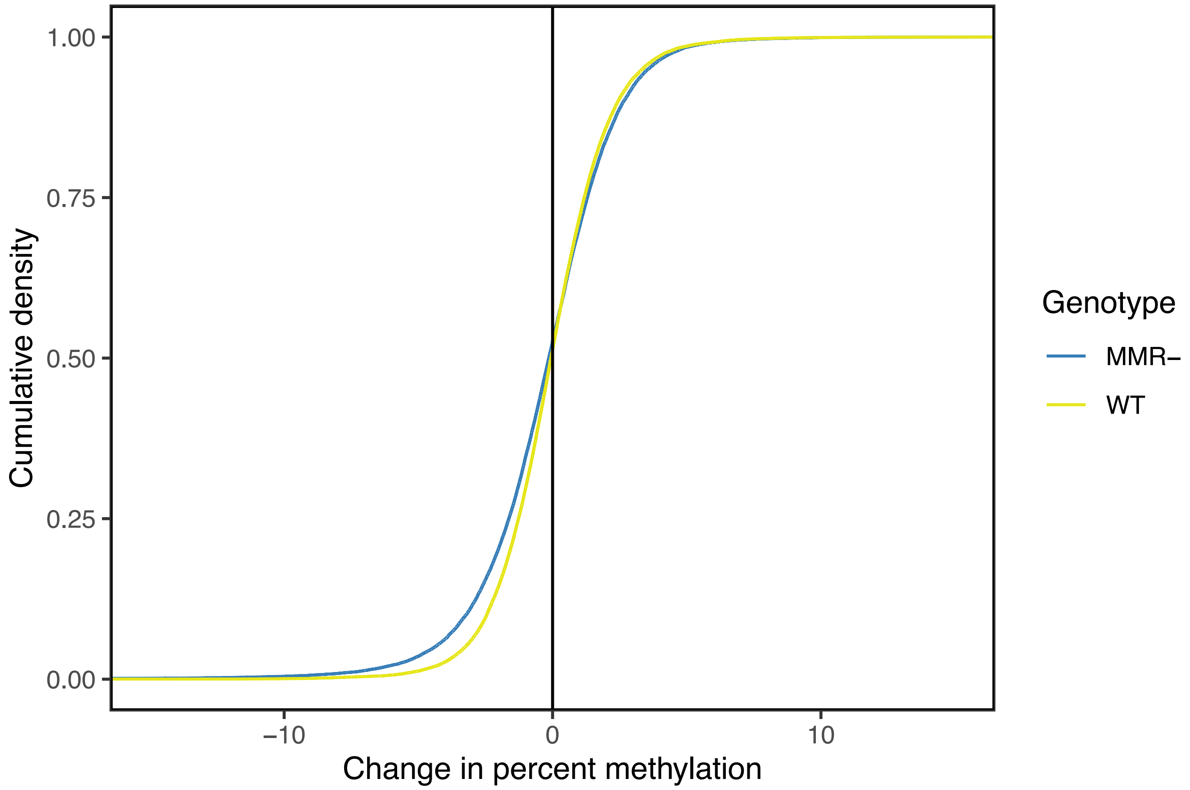

Figure S8. Distribution of changes in per-site methylation between evolved genotypes and their isogenic ancestor. For each GATC site shared across all the evolved clones and the ancestral strain, the difference between average percent methylation in the evolved genotypes and in the ancestor replicates were calculated, and values were used to calculate empirical cumulative density functions. Evolved MMR- clones (blue) exhibit a left-shift indicating a genome-wide bias towards reduced methylation compared to evolved WT clones (yellow).
